# Boston Letter

**Published:** 1880-08

**Authors:** 


### Domestic Correspondence.

---

### ARTICLE X.

### BOSTON LETTER.

*Messrs. Editors:*—At this season of the year you cannot reasonably look for medical news of such freshness as the breezes we would welcome if we could find them. Our Canadian weather prophet has been so desperately exact in his prognostications, that we have not only had the torrid " spell " he so confidently predicted, but weather of such intensity that Dante's Inferno is nothing to it. Strangely enough, however, sickness does not seem to be very rife. The wealthy have gone to the sea and the hills. The poor live out of doors, and the blessed agency of constant inhalation of pure air, the ease with which, for one horse-car fare, they can reach City Point, half way down the bay, and the charitable picnics and steamer excursions for the children, all have their influence in keeping, and even renewing, the health of these city-bound people. We have our share of sunstrokes, but the sufferers being for the most part men who are intemperate, we feel that the effect of the sun upon them is pretty generally their fault. It is rather odd that the east wind, so disagreeable in early spring and such a beneficent guest in the warmer days, has for the past three weeks, our heated term, almost literally been absent. The prevailing winds have come from the south, and our sufferings have thereby been equal to those of Charlestonians, in whose city, one day this week, the mercury ranged two or three degrees below our figures.

Last winter I quoted the charitable remarks of a member of our Suffolk District Society (not "Historical Society," as your types made me say), in relation to the proposal of the faculty of

Harvard Medical School to erect a new college building. I have remarked, by the way, that this gentleman's opinion of the faculty, all unjust and unreasonable as it was, has been quoted from my letter by Western journals without the context which should have given it its proper value. When I wrote the letter to which I refer, it was supposed that the ground occupied by our old Cochituate reservoir was the chosen site of the new building. The faculty of the school, however, have done a wiser thing. The corporation of the University has secured from the State of Massachusetts the westerly half of a square on what we term the "back-bay lands." It contains 33,000 square feet, and being much more centrally located than the present building, will be far easier of access. Moreover the new structure will stand about half way between the City and Massachusetts General hospitals. For teachers and students this will be a great advantage in the saving of time. It will also be an ornamental addition to the fine buildings, the old South Church, the Museum of Fine Arts, Trinity Church, the buildings of the Boston Society of Natural History and the Institute of Technology, which stand in close neighborhood. The Medical Library on Boylston place and the Public Library, which contains a fine collection of medical works, will also be in close proximity. Many of the students of the literary department of Harvard University from time to time attend certain of the clinics and lectures of the Medical School. So soon as the new bridge over Charles river is completed they can more quickly reach the new building than the old. It is thought that the centennial celebration of the foundation of the Medical School (1782), will occur in the new structure. It will be worthy of its surroundings and its accomodations will fully meet the needs of the constantly increasing classes which avail themselves of the advantages of this school. No professional department of the University has enjoyed a more prosperous career than has the Medical School. Hardly a medical specialty can be named that cannot be studied here. The corps of instructors has been much enlarged and the ground now covered requires so much time that the faculty have been led to announce an important change in the plan of study. The present curriculum has received several years of fair trial, and has proved a gratifying success. But in spite of the increased amount of time given to the course, it was soon found that three full academic years were insufficient to complete the various branches of instruction. For this reason the faculty have matured a plan—the announcement of which you may have seen—a plan which covers four years, and at present is elective. Matriculates who do not choose to make use of it can adopt the old three years' course which, as yet, has not been altered in any respect. It might have been wiser on the part of the faculty if the new plan had been announced as obligatory. Such a decision would have met with warm approval and undoubtedly would have proved a success. The new plan will go into operation at the beginning of the next session. For details of the course I must refer you to the prospectus of the school. I will merely add that such students as follow the three years' plan will receive their diplomas as usual, while such as adopt the new course, complete four full years of study and obtain an average of 75 per cent. in all the examinations, will take the degree of Doctor of Medicine *cum laude.* Another refinement has been made in increased stringency of the preliminary examination. In order to be allowed to matriculate, a candidate must pass an examination in English composition and dictation, must translate easy Latin prose, have a complete knowledge of physics and show familiarity with either French, German, elments of Algebra or of plain Geometry, and Botany. These are elective branches.

When it is remembered that the new course of study is the minimum of that exacted in Europe, one cannot see why it should not be welcomed by all ambitious students. It entails self-exertion and self-discipline which will lay a solid foundation for a successful future.

It should be known that at the Harvard Medical School a student can now acquire an education which will preclude the necessity of European study. This will afford valuable advantages to men who have not the means to go abroad. There are teachers who are strong opponents of medical study in Europe. They say it is unnecessary for Americans to leave this country to obtain a professional education. In one sense this is true, for all requisite knowledge can be obtained here. But a broader opinion would be that a year or two spent in European clinics and hospitals would give a student greater breadth of view and more liberality of thought; he widens the scope of his mind by placing himself under the teaching of men whose mental influence is far different from that of American teachers. Moreover, he sees an immense quantity of clinical material, and becomes familiar with cases which he might not meet in the more limited number of patients seen at home. He becomes familiar with one or two new languages, and likewise with certain specialties which are more thoroughly taught in the complete splitting up of medicine in Europe, than in this country. In short, he not only becomes more finely educated, but acquires a politer culture than if he went into practice directly from an American school. I have always thought, however, that a Continental course of instruction may be made much more useful in a practical sense, if the student has already had sufficient experience in practice at home to have discovered just what he especially needs. He can then make a wiser use of his time while abroad. Desultory use of clinics and hospitals is not beneficial. The student must have a plan and follow it without deviation.

The annual meeting of the Massachusetts Medical Society, held a month since, was remarkably uneventful. The Councilors' session usually develops human nature to a marked degree; but on this occasion everything was calm and placid. No questions of burning interest were brought up. The election of officers, however, was made peculiarly interesting by the fact that the next annual meeting of the society will also be its centennial anniversary. The choice of president, orator and annual chairman (who presides at the dinner), was made with direct reference to the nature of the meeting. Dr. H. W. Williams was made president, Dr. J. Collins Warren orator, and Dr. J. C. White annual chairman. The only ripple at the recent meeting was caused by a very energetic Fellow, who laid the wires for the reintroduction of the woman question next year.

You have heard of the attempt last spring to pass, through the legislature of this State, a bill regulating the practice of medicine. The bill, if it had passed, would have disposed of our horde of quacks and charlatans. It was defeated, and the genus quack still holds undisputed sway. When the proposed law was first made known, it met with an earnestness of welcome which made it seem a success. Few would have dared wager that it would be defeated. It received the sanction of most of our leading physicians. But a minority opposed it strongly on the ground that it gave too much recognition to homœopaths and eclectics, each of whose schools were to be represented on the proposed State board of examiners. This opposition won friends so rapidly that pretty soon the bill seemed to have but few supporters. Even those who favored it, many of them, turned against it, and it failed. We are annoyed and harassed by quacks. They exist in large numbers. Of course the physicians and all well-minded citizens wish to drive them from the State. Sooner or later there will be drafted a bill which will rid us of this outrageous incubus. * *

BOSTON, July 18, 1880.

---

CHRONIC URETHRITIS TREATED BY INJECTIONS OF WATER SATURATED WITH TINCTURE OF IODINE.—Dr. Masurel. *Bull. Méd. du Nord.* and *Revue Médicale*, Jan. 3, 1880, p. 20.—Two injections are made the first day with an interval of 12 hours between. The next day there is but slight discharge, and a second injection may be given or not; the third day a new injection should be given and the patients are cured. Exceptionally a fourth injection has been given, but if it does not succeed something else should be tried.

POMADE FOR THE SPOTS OF PREGNANCY.—Neumann. *L'Union Médicale*, No. 59.—Chrysophonic acid, 1 gram; Lard, 40 grams; mix. The skin is cleaned with soap and water and then annointed with the ointment, a cloth being laid over to prevent it from running. Three or four applications are made, at intervals of two days, care being used to avoid the eyelids and to not use too strong an ointment for delicate skins. The regions covered become red, then black; the skin desquamates and the spot disappears.

